# *Lin28B* Is an Oncofetal Circulating Cancer Stem Cell-Like Marker Associated with Recurrence of Hepatocellular Carcinoma

**DOI:** 10.1371/journal.pone.0080053

**Published:** 2013-11-14

**Authors:** Shu-Wen Cheng, Hung-Wen Tsai, Yih-Jyh Lin, Pin-Nan Cheng, Yu-Chung Chang, Chia-Jui Yen, Hsuan-Pang Huang, Yun-Pei Chuang, Ting-Tsung Chang, Chung-Ta Lee, Anning Chao, Cheng-Yang Chou, Shih-Huang Chan, Nan-Haw Chow, Chung-Liang Ho

**Affiliations:** 1 Institute of Basic Medical Sciences, College of Medicine, National Cheng Kung University, Tainan, Taiwan; 2 Department of Pathology, National Cheng Kung University Hospital, College of Medicine, National Cheng Kung University, Tainan, Taiwan; 3 Graduate Institute of Clinical Medicine, College of Medicine, National Cheng Kung University, Tainan, Taiwan; 4 Infectious Disease and Signaling Research Center, National Cheng Kung University, Tainan, Taiwan; 5 Department of Surgery, National Cheng Kung University Hospital, College of Medicine, National Cheng Kung University, Tainan, Taiwan; 6 Department of Internal Medicine, National Cheng Kung University Hospital, College of Medicine, National Cheng Kung University, Tainan, Taiwan; 7 Department of Medical Laboratory Science and Biotechnology, College of Medicine, National Cheng Kung University, Tainan, Taiwan; 8 Department of Ophthalmology, Chang Gung Memorial Hospital, Linkou and Taipei, Taiwan; 9 College of Medicine, Chang Gung University, Taoyuan, Taiwan; 10 Department of Obstetrics and Gynecology, National Cheng Kung University Hospital, College of Medicine, National Cheng Kung University, Tainan, Taiwan; 11 Department of Statistics, National Cheng Kung University, Tainan, Taiwan,; National Cancer Institute, United States of America

## Abstract

By using an expressed sequence tag bioinformatic algorithm, we identified that *Lin28 homolog B* (*Lin28B*) may have an oncofetal expression pattern which may facilitate detecting cancer cells in adults. It is also reported to be a potential marker for cancer stem cells. Therefore, we sought to verify oncofetal-stemness characters of *Lin28B* and test its potential as a circulating cancer stem cell-like marker in adult HCC patients. *Lin28B* mRNA was examined in a panel of fetal tissue, adult tissue and tumors. *Lin28B* was over-expressed or knocked down in HepG2 cells to evaluate its potential as a stem cell-like marker. RT-qPCR for *Lin28B* was performed in the peripheral blood mononuclear cells from patients with HCC receiving surgery (n=96) and non-HCC controls (n=60) and analyzed its clinical significance. *Lin28B* showed an oncofetal expression pattern. Its overexpression could upregulate stemness markers (OCT4, Nanog and SOX2) and enhance tumorsphere formation *in vitro*. *Lin28B* knockdown had opposite effects. Circulating *Lin28B* was detected in peripheral blood mononuclear cells in 3 cases (5%) of non-HCC controls and 32 cases (33.3%) of HCC patients. In HCC patients, circulating *Lin28B* was associated with high tumor grade (P=0.046), large size (P=0.005), high AJCC stage (P=0.044) and BCLC stage (P=0.017). Circulating *Lin28B* was significantly associated with decreased recurrence-free survival (P<0.001). Circulating *Lin28B* separated early stage HCC into 2 recurrence-free survival curves (P=0.003). In multivariate analysis, circulating *Lin28B* was an independent variable associated with early recurrence (P=0.045) and recurrence in early stage HCC (P=0.006). In conclusion, the oncofetal gene *Lin28B* is a potential oncofetal cancer-stem-cell-like circulating tumor cell marker that correlates with HCC recurrence after hepatectomy. Circulating *Lin28B* could refine early AJCC stages. Our finding supports the possible use of a TNMC (C for circulating tumor cells) staging system in HCC.

## Introduction

Hepatocellular carcinoma (HCC) is a major cause of cancer death in Taiwan and one of the most common cancers worldwide [1]. Therefore, it is imperative to identify biomarkers in predicting the development of HCC and clinical outcome. Alpha-fetoprotein (AFP) and glypican-3 are current tumor markers for HCC. Both of them belong to oncofetal genes which are defined as genes expressed in the embryos or fetuses, turned off or suppressed in adult tissue, but re-expressed in tumor cells[2,3]. Oncofetal genes/proteins tend to be good tumor markers due to low background expression in the adults. In a previous study, we used a combined bioinformatic and experimental approach to search for new oncofetal genes [4]. We categorized 6118 expressed sequence tag (EST) libraries into 3 groups: immature (n=483), mature (n=1724), and tumor (n=3911). By using the calculated frequencies of the AFP gene in each group as references to set the thresholds of bioinformatics analysis, we successfully identified 44 unknown genes with potential oncofetal expression patterns. One of the genes, LRRC16B was further studied [4]. The result supports that this bioinformatic algorithm can bring out oncofetal genes with important functions. Also present in our gene list was the gene *Lin28 homolog B* (*Lin28B*), which was unknown at that time. The calculated frequencies of *Lin28B* gene in the libraries of immature, mature, and tumor groups were 22, 5 and 28, respectively, with ratio between tumor and mature groups (tumor/mature) estimated at 5.6 (Table S1). 

 The mammalian homologs of lin-28, *Lin28* and *Lin28B*, are microRNA binding proteins. They regulate let-*7* by binding to the terminal loop of *let-7* family miRNA precursors and block their processing into mature miRNAs, resulting in derepression of *let-7* targets, such as *K-ras* and *c-Myc* [5,6]. *Lin 28* is highly expressed in embryos and embryonic stem cells [7]. It can reprogram human somatic fibroblasts to pluripotency when co-expressed with *OCT4*, *Nanog* and *SOX2* [8]. Thus, *Lin28* may involve in the embryonic development and maintenance of embryonic stem cells. As for *Lin28B*, it is able to promote cell proliferation and transformation, but its function in embryonic development remains unclear [9-11]. *Lin28B* is frequently expressed in HCC and is associated with poor patient prognosis, as compared to *Lin28* [9-13]. Since *Lin28* is known to be a marker for stem cells, we predicted *Lin28B* is also related to cellular stemness and is potentially a marker for cancer stem cells. Such a notion was supported by recent publications in which *Lin28B* was shown to be associated with the stemness of prostate and colon cancer cell lines [14,15]. 

 Reverse transcription quantitative real-time PCR (RT-qPCR) of Ficoll-separated cells or buffy coat has been used to detect tumor cell transcript as a surrogate for detecting circulating tumor cells (CTCs) [16-20]. To decrease the background level and increase the differentiation power, the ideal markers should have low likelihood to be expressed in white blood cells or endothelial cells. It would be even better if they are not expressed in any adult tissue at all. Therefore in theory, the oncofetal genes should be good tumor markers in peripheral blood samples. Since *Lin28B* is a candidate oncofetal gene which is possibly related to stem cell phenotypes, it is a potential surrogate tumor marker to detect circulating cancer stem cells which have been shown to have highly predictive value for cancer recurrence and metastasis [21,22].

 Therefore, the purpose of this study was to verify the dual oncofetal and cancer-stem-cell characteristics of *Lin28B* and to evaluate its clinical significance when detected in circulating cells in HCC patients. The hypothesis tested was that circulating *Lin28B* detected in peripheral blood mononuclear cells is an oncofetal cancer-stem-cell-like marker associated with recurrence or worse survival in HCC.

## Materials and Methods

### Tumor cell lines

 The cell lines used in this study were human hepatic (Huh-7, HepG2 and PLC/PRF/5), ovarian (PA-1, TOV-21G, SK-OV-3, BG-1, NIH:OVCAR-3, and ES-2), renal (786-O and ACHN), bladder (T24 and TSGH-8301), breast (MCF-7), pulmonary (A549), and colon (SW480) cancer cell lines. Huh-7 was obtained from JCRB. HepG2, PLC/PRF/5, PA-1, TOV-21G, NIH:OVCAR-3, ES-2, MCF-7, SW480, T24 and TSGH-8301 were obtained from BCRC. A549 was obtained from ATCC. SK-OV-3 and BG-1 were a kind gift from Prof. Tzu-Hao Wang[23]. 786-O and ACHN were a kind gift from Prof. Yeong-Shiau Pu[24]. A549, NIH:OVCAR-3, SK-OV-3, and BG-1 were maintained in DMEM/F12. T24, TSGH-8301, Huh-7, HepG2, MCF-7 and SW480 were maintained in DMEM. 786-O and ACHN were maintained in RPMI1640. PLC/PRF/5 and PA-1 were maintained in MEM. ES-2 was maintained in McCoy’s 5a. TOV-21G was maintained in MCDB105 and M199 (1:1). All media were supplemented with 10% fetal bovine serum, 100U/ml penicillin and 100mg/ml streptomycin (Gibco, Grand Island, NY,USA) under 5% CO2 at 37°C.

### The cDNA libraries and paired tumor and non-tumor tissue samples

 The fetal cDNA libraries purchased from BioChain (Hayward, CA, USA) were compared with normal human adult cDNA libraries purchased from Clontech (Becton-Dickenson, Franklin Lakes, NJ). Paired tumor and non-tumor tissue from various organs was collected from patients admitted for surgery in National Cheng Kung University Hospital, Tainan, Taiwan. 

### Magnetic cell sorting to enrich EpCAM+ cells

 EpCAM-positive PLC/PRF/5, Huh-7 and HepG2 cells were isolated by magnetic cell sorting (MCS) using monodispersed magnetizable particles according to the manufacturer’s instructions (CELLection^TM^ Pan Mouse IgG kit) using anti-EPCAM antibody (Epitomics, Burlingame, CA). EpCAM^+^ and EpCAM^-^ PLC/PRF/5, Huh-7 and HepG2 cells were lysed for western blot assays.

### Retroviral Infection

 We used a retrovial system to overexpress *Lin28B* in the HCC cell lines. pMSCVpuro (BD Clontech), pMSCV-LIN28B and pSUPERretro vectors were co-transfected into GP2-293T package cells with VSV-G plasmids using the calcium phosphate method for 48 h. The HepG2 cell was seeded in 1×10^6^ cells per well in a 6-cm dish and incubated overnight under 5% CO_2_ at 37 °C. Retroviral supernatant was added with 8 ng/ml of polybrene (Sigma, St Louis, MO, USA), and used to infect HepG2 cell. Pooled HepG2 cell populations expressing either pMSCVpuro or pMSCV-LIN28B were selected with 0.7μg/mL of puromycin (Sigma-Aldrich, St Louis, MO).

### shRNA lentivirus production

 We used a lentiviral shRNA system to knock down *Lin28B* in the HCC cell lines. pLKO.1 plasmids expressing small hairpin RNA (shRNA) were purchased from the National RNAi Core Facility (Academia Sinica, Taipei, Taiwan). The lentivirus particles were obtained from the RNAi Core, the Research Center of Clinical Medicine, National Cheng Kung University Hospital. To knock down Lin28B expression, the shRNA of *Lin28B* (TRCN0000219860, target sequence: 5'- CATAACAGGTCTTCTTCATAT-3') was adopted. A plasmid pLKO_TRC005 was used as the negative control.

### Western blotting

 Collected cells were dissolved by lysis buffer (Complete Lysis M, EDTA free, Roche) on ice and then centrifuged at 10000 xg, 4°C for 20 minutes. Then, 100μg of protein was loaded and separated by 12 % SDS-PAGE, and transferred to a polyvinylidene fluoride membrane (Millipore, Billerica, MA, USA). Lower amount of protein (40μg) was loaded in the magnetic cell sorting assay due to lower total cell number collected. Primary antibodies included rabbit anti-Lin28B (Cell signaling technology), mouse anti-OCT4 (Santa Cruz Biotechnology, Santa Cruz, CA), rabbit anti-Nanog (Epitomics, Burlingame, CA), rabbit anti-SOX2 (Epitomics, Burlingame, CA), rabbit anti-EpCAM (Epitomics, Burlingame, CA) and mouse anti-β-actin (Millipore). 

### Sphere formation assay

 Sphere formation assay was performed as previously described[25]. Briefly, HepG2 cells were seeded on uncoated 6-well culture plates (BD Labware, Bedford, MA) in DMEM/F-12 serum-free medium (caisson) contained 1% MEM NEAA, 1X N2, 20 ng/ml EGF, 10 ng/ml bFGF, 100 μg/ml penicillin G, and 100 U/ml streptomycin (Invitrogen, Grand Island, NY). After 9 day culture, wells were examined under an inverted microscope at x20 magnification, and the number of spheres of >50 μm in diameter were counted under a light microscope.

### Patients, samples and clinical data

 One hundred and nineteen patients who had primary HCC underwent hepatectomy at National Cheng Kung University Hospital from January 2006 through December 2011, were included. Pre-surgery whole blood samples were sent for peripheral blood mononuclear cell collection. Patients with a previous diagnosis of cancer, distant metastasis before hepatectomy, positive surgical margins, a diagnosis of combined hepatocellur-cholangiocarcinoma (CHC), or poor sample RNA quality were excluded. Ten patients were excluded due to a positive surgical margin or a diagnosis of CHC and 13 patients were excluded due to poor sample RNA quality. The remaining 96 patients were included for further study (Figure S1). The follow-up interval was every 3 months. Recurrence of HCC was documented upon typical findings of computed tomography or magnetic resonance imaging with or without raised serum AFP level or pathological confirmation. Recurrence-free survival (RFS) was defined as time from surgery to the first occurrence of either local or distant recurrence. Disease-specific survival (DSS) was defined as time from surgery to HCC-related death. Subjects were censored at the last follow-up appointment or at death without recurrence. The patient profiles of the 96 HCC patients were shown in Table S2. The median duration of follow-up was 19.7 months (range, 0.1-41.5 months). Forty patients (41.7%) had recurrent HCC (median duration until recurrence, 7 months; range, 1.5-31.6 months), including local recurrence in 32 patients, metastasis in 5 patients, and both local recurrence and metastasis in 3 patients. Ten patients (10.4%) died of HCC (median survival, 13.8 months; range, 1.6-21.9 months). For comparison, 60 individuals without HCC (non-HCC group) were also included: 31 healthy individuals without a liver disease and 29 patients with viral hepatitis, including 8 patients with cirrhosis (16 HBV and 13 HCV). The mean age of the healthy individuals without liver disease was 44.1 years (range, 20-75 years; 10 men and 21 women), and of the patients with hepatitis was 49.4 years (range, 24-67 years; 20 men and 9 women).

 Informed consent in writing was obtained from each patient and the study protocol conformed to the ethical guidelines of the 1975 Declaration of Helsinki as reflected in a priori approval by the Human Experiment and Ethics Committee of National Cheng Kung University Hospital in Tainan, Taiwan.

### Peripheral blood sample preparation

 Whole blood samples were collected in 10-ml pyrogen-free tubes containing 0.12 ml of K_3_EDTA 15% (BD Vacutainer K_3_EDTA; Becton Dickinson, Franklin Lakes, NJ) and then layered on equal volumes of Ficoll-Hypaque density gradient (Histopaque-1077; Sigma-Aldrich, Taufkirchen, Germany). Peripheral blood mononuclear cells were recovered from the interphase. The cell pellets were washed and collected for subsequent RNA extraction.

### RNA extraction

 Total RNA of primary tissue and cell lines were extracted using the Trizol reagent (Life Technologies, Carlsbad, CA). The RNA of blood samples was extracted using a kit (QIAamp RNA Blood Mini Kit; Qiagen) according to the manufacturer’s protocols.

### Reverse transcription polymerase chain reaction (RT-PCR) and Reverse transcription quantitative real-time PCR (RT-qPCR)

 2 μg RNA was reversely transcribed using SuperScript II (Invitrogen, Grand Island, NY). The semiquantitative RT-PCR primer sequences were shown in Table S3. *β-actin* was used as a internal control gene in RT-PCR. The cDNAs was subjected to RT-qPCR using a LightCycler system (Roche, Mannheim, Germany). For RT-qPCR in various fetal and normal adult organs and blood samples, the levels of *Lin28B* were normalized to *GAPDH* which has been used as a reference gene in detecting circulating tumor cells [19,26]. For liver and HCC tissue samples, the levels of *Lin28B* were normalized to *tyrosine 3-monooxygenase/tryptophan 5-monooxygenase activation protein, zeta polypeptide* (*PLA*) instead of *GAPDH* because there was a wide range of variation in the expression levels of *GAPDH* among the liver and HCC tissue samples. *PLA* had a medium expression level in liver tissue [27] and its expression levels were more stable among HCC tissue samples. Both RT-qPCR primers and probes were purchased (Applied Biosystems; sequences not shown) or synthesized as designed by LightCycler Probe Design Software 2.0. Both the fluorescent TaqMan probes and the designed amplification primer sequences are listed in Table S4. Each standard curve was calculated with serial dilutions (10^0^-10^6^ copies) of plasmid templates. The threshold cycle (Ct) of samples was converted into the copy number of the mRNA using standard curves derived from serial dilutions of constructs of *Lin28B* and *GAPDH*, respectively. Nucleic acid extracted from HepG2 cells was used as a positive control. A negative control in which template total RNA was replaced by sterile water was included. The dynamic range of the RT-qPCR for *Lin28B* was from 10 to 10^6^ copies of plasmid templates (Figure S2). A Ct value less than 38 and greater than zero indicates positive expression of *Lin28B* gene, whereas a Ct value equal to or greater than 38 or no Ct value indicates negative expression of *Lin28B* gene. 

### Statistical analysis

 The difference in tumorsphere formation between *Lin28B*-expressing and -knockdown cell lines was tested for significance using student's *t*-test. *Lin28B* mRNA expression in peripheral blood was compared between groups using Wilcoxon rank sum test and was correlated with clinicopathological indicators using chi-square test or Fisher’s exact test. Survival curves and probabilities were estimated using the Kaplan-Meier method, and the log-rank test was used to assess the significance of difference between groups. A leave-one-out cross validation was used to determine a cutoff value associated with survival. Univariate and multivariate Cox proportional hazards regression model was used to determine the significance of different prognostic factors. The multivariate analyses were adjusted simultaneously for age, sex, type of viral infection, cirrhosis, tumor grade, status of multifocal tumors, satellite nodules, vascular invasion, tumor size, ***AmericanJointCommitteeonCancer*** (AJCC 7th edition) stage, Barcelona-Clinic Liver Cancer (BCLC) stage and serum AFP. Statistical significance was set at *P* < 0.05. The analysis of peripheral blood samples from 96 patients will have 80% power to detect a hazard ratio of 2.2 between *Lin28B* (+) and *Lin28B* (-) HCC patients. The proportional hazards assumptions were checked by the martingale and deviance diagnostic plots and no significant deviation from the assumptions of the proportional hazard regression model existed.

## Results

### The oncofetal characteristics of Lin28B

**Figure 1:**
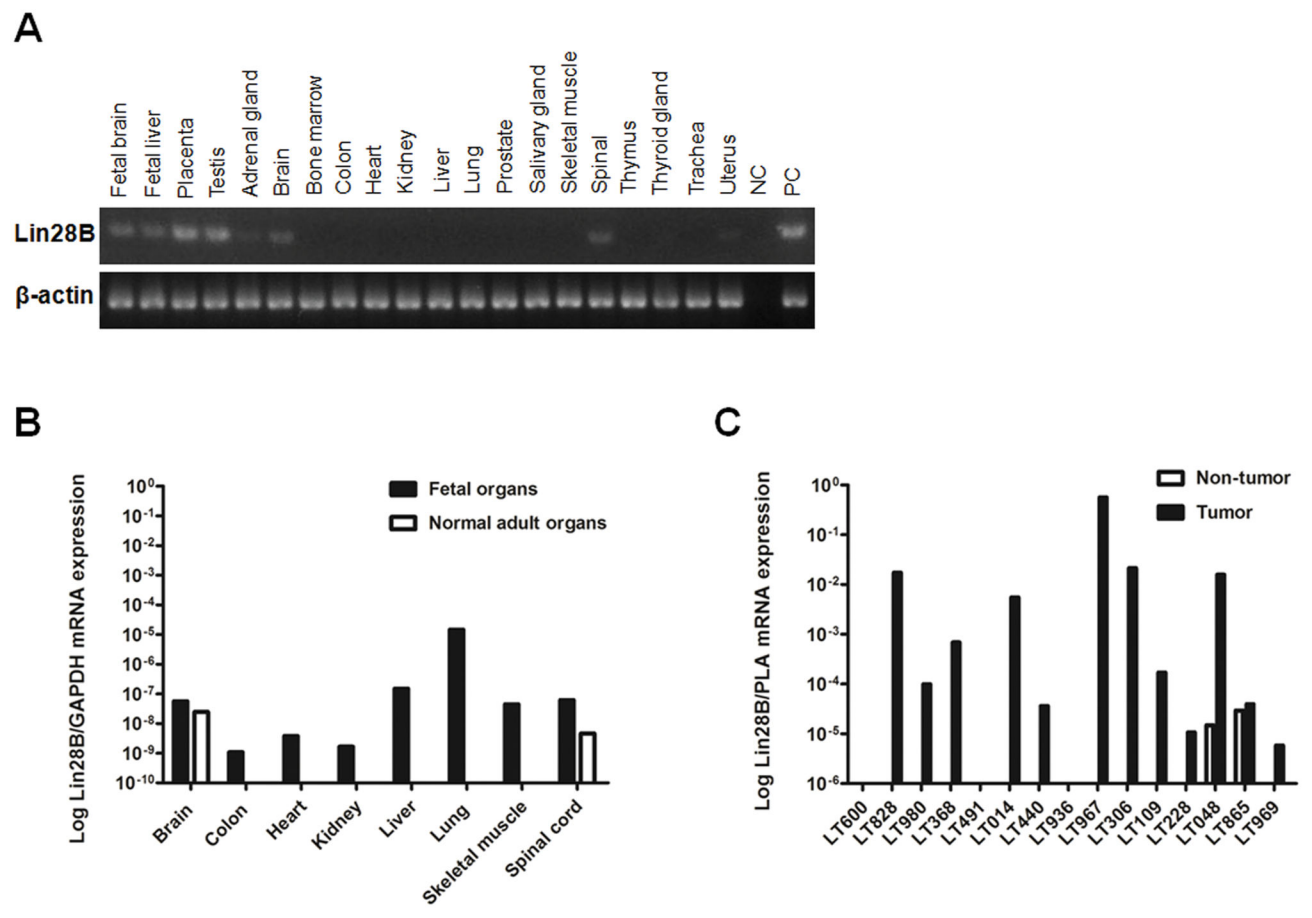
The oncofetal expression profile of *Lin28B*. **A**, RT-PCR showed that *Lin28B* was expressed in fetal brain and liver and in adult testis, brain, spinal cord and placenta. It was not detected in other adult tissues. **B**, RT-qPCR showed that *Lin28B* was markedly downregulated from fetal (closed bar) to adult (open bar) tissues except for the brain. **C**, RT-qPCR was performed using pairs of HCC (closed bar) and non-tumor (open bar) liver tissues. Overexpression of *Lin28B* (> 100×) was observed in 8 HCC samples (53.3%). NC, negative control; PC, positive control.

 To test whether *Lin28B* is an oncofetal gene, its expression was examined in a panel of human total RNA (Master Panel II, BD Clontech) using RT-PCR (Figure 1A). *Lin28B* was expressed in fetal brain, fetal liver, normal adult placenta, testis, brain, and spinal cord. It was not expressed in other adult tissues. The change of expression from fetal to adult tissues was quantitated by RT-qPCR using total RNA selected from Human Fetal Normal Tissue (BioChain) and Master Panel II (BD Clontech) tissue panels (Figure 1B). *Lin28B* was expressed in the fetal organs, but was significantly down-regulated in their adult counterparts, except for the brain.

For screening possible *Lin28B* expression in different tumor types, RT-PCR was performed in various human cancer cell lines. *Lin28B* could be detected in the ovarian, hepatic, and colorectal cancer cell lines (Figure S3). To examine its expression in tumors, RT-PCR was performed on five cases of paired tumor and non-tumor tissues from 5 types of common cancers (Figure S4). *Lin28B* was overexpressed in some of the tumors from breast (1/5), uterus (1/5), lung (4/5), liver (1/5), and ovary (1/5), but was expressed at very low levels in the non-tumor tissue. RT-qPCR for *Lin28B* expression was further performed on 15 HCC tumor and non-tumor tissue pairs (Figure 1C). Over-expression of *Lin28B* (> 100×) was observed in 8 tumor tissue samples (53.3%). These results confirmed the oncofetal nature of *Lin28B*. Two non-tumor liver tissue samples (13%) showed low level of *Lin28B* expression. Microscopically, one of these two samples showed cirrhosis with focal large cell change and the other showed chronic hepatitis with mild interface activity. The histological findings showed no obvious difference from those of the 13 non-tumor liver tissue samples without *Lin28B* expression, in which seven cases showed cirrhosis and 6 cases showed chronic hepatitis with interface activity. Four cases showed large cell change and one case showed small cell change.

### Elevated expression of Lin28B in EpCAM-enriched stem cell-like population

**Figure 2:**
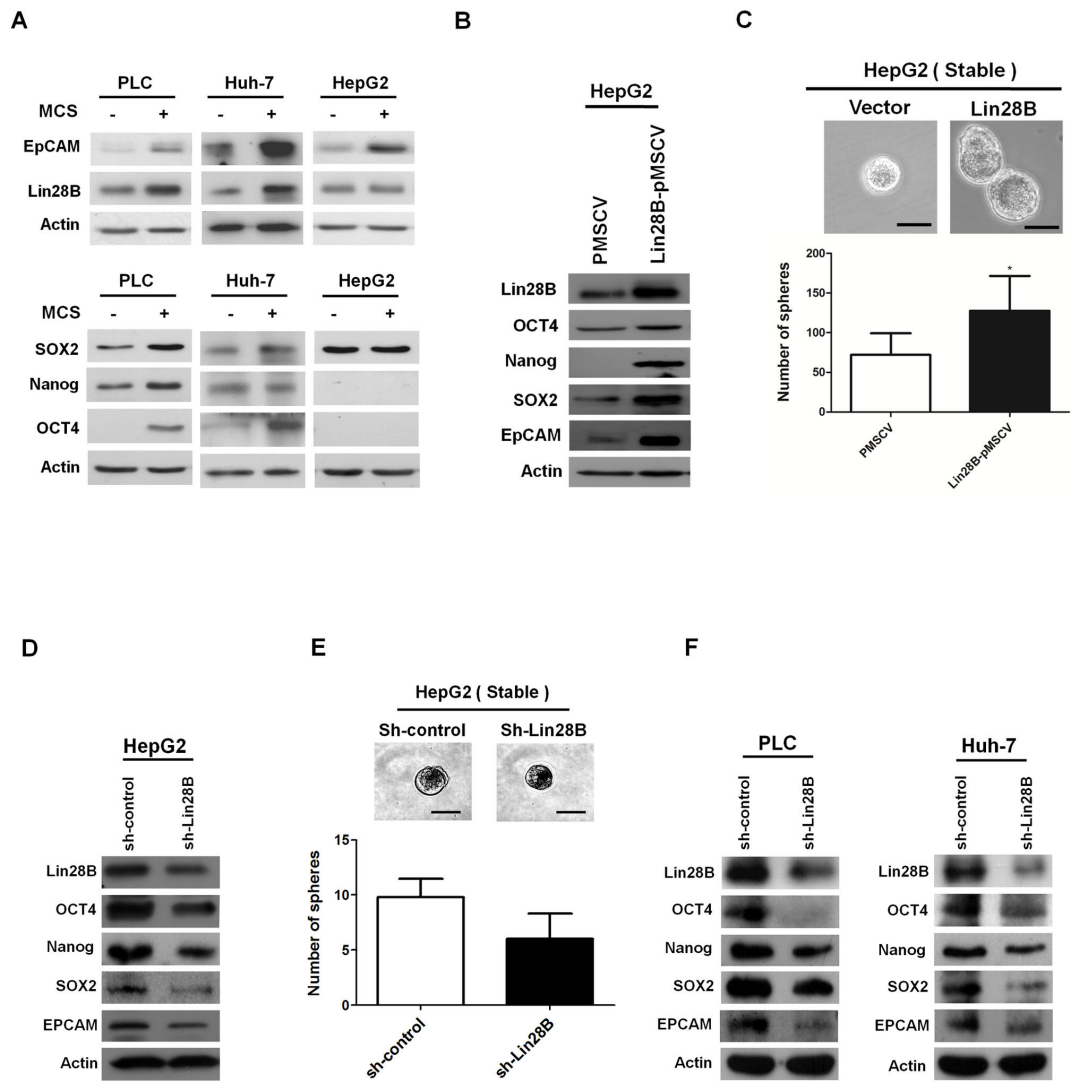
Expression of Lin28B associated with stemness markers in HCC cells. Western blot analyses showed that Lin28B was overexpressed in captured EpCAM^+^ PLC/PRF/5 cells and Huh-7 cells but not in EpCAM^+^ HepG2 cells (A, upper panel). EpCAM^+^ PLC/PRF/5 cells demonstrated elevated levels of SOX2, Nanog and OCT4; EpCAM^+^ Huh-7 cells, SOX2 and OCT4; but EpCAM^+^ HepG2 cells, none (A, lower panel). Lin28B-pMSCV HepG2cells showed increased levels of OCT4, Nanog, SOX2 and EpCAM in western blot (**B**) and formed more spheres than vector control cells (P=0.032) (**C**). sh-Lin28B HepG2 cells showed decreased levels of OCT4, Nanog, SOX2 and EpCAM in western blot (**D**) and tended to form fewer spheres than vector control cells (P = 0.059) (**E**). sh-Lin28B PLC/PRF/5 cells and Huh-7 cells also decrease expression of stem cell markers (**F**). Lower amount of protein (40μg) was loaded in the MCS assay than that (100μg) loaded in the *Lin28B*-overexpression assay due to lower total cell number collected in MCS assay. Different viral systems were used in the experiments: retrovirus in *Lin28B* over-expression assay and lentivirus in *Lin28B* knock-down assay. Cell growth was slower when infected with lentivirus. MCS, magnetic cell sorting. Scale bar in C and E, 50 μm.

 EpCAM is a surface marker that was reported to be able to enrich the hepatic stem cell-like population [28]. Therefore, to investigate whether *Lin28B* is expressed in cancer stem cells, we performed magnetic cell sorting (MCS) to separate HCC cell lines into EpCAM^+^ and EpCAM^-^ populations. The protein level of Lin28B was increased in EpCAM^+^ PLC/PRF/5 cells and Huh-7 cells (Figure 2A, upper panel), along with increased levels of stem cell markers, SOX2, Nanog and OCT4 in PLC/PRF/5 cells and SOX2 and OCT4 in Huh-7 cells (Figure 2A, lower panel). In contrast, EpCAM-capture did not enrich the stem cell-like population in HepG2 cells, since there was no difference of SOX2, Nanog, or OCT4 expression between EpCAM^+^ and EpCAM^-^ HepG2 cells (Figure 2A, lower panel). Lin28B also showed no difference between these two populations (Figure 2A, upper panel). Taken together, our findings supported the notion that Lin28B is expressed in cancer stem cell-like subpopulations.

### Increased in vitro stemness with Lin28B overexpression

 Because EpCAM could not enrich the *Lin28B*-expressing stem cell-like population in HepG2 cells, we established a *Lin28B*-overexpression HepG2 stable pool by retrovirus infection to investigate the effect of Lin28B on stem cell-like phenotypes. Representative stem cell markers were analyzed by Western blotting. In comparison to the vector control, Lin28B overexpression upregulated the stem cell markers: SOX2, Nanog, and EpCAM. A mild increase of OCT4 was also observed (Figure 2B).

To determine the effect of *Lin28B* on tumor sphere-formation, both *Lin28B*-expressing and control cells were cultured in suspension to generate spheres as an indicator of a cancer stem-like property *in vitro* [25,29,30]. As shown in Figure 2C, *Lin28B*-expressing HepG2 cells showed an increased tumorsphere forming ability compared with the vector control cells (P=0.032).

On the contrary, knocking down *Lin28B* in HepG2 cell line downregulated the expression of OCT4, Nanog, SOX2 and EpCAM (Figure 2D) and tended to reduce the tumorsphere formation (P=0.059) (Figure 2E). Furthermore, knocking down *Lin28B* in PLC/PRF/5 cells and Huh-7 cells also downregulated the expression of the stem cell markers (Figure 2F).

### Detection of Lin28B mRNA in the peripheral blood cells of HCC patients

**Figure 3:**
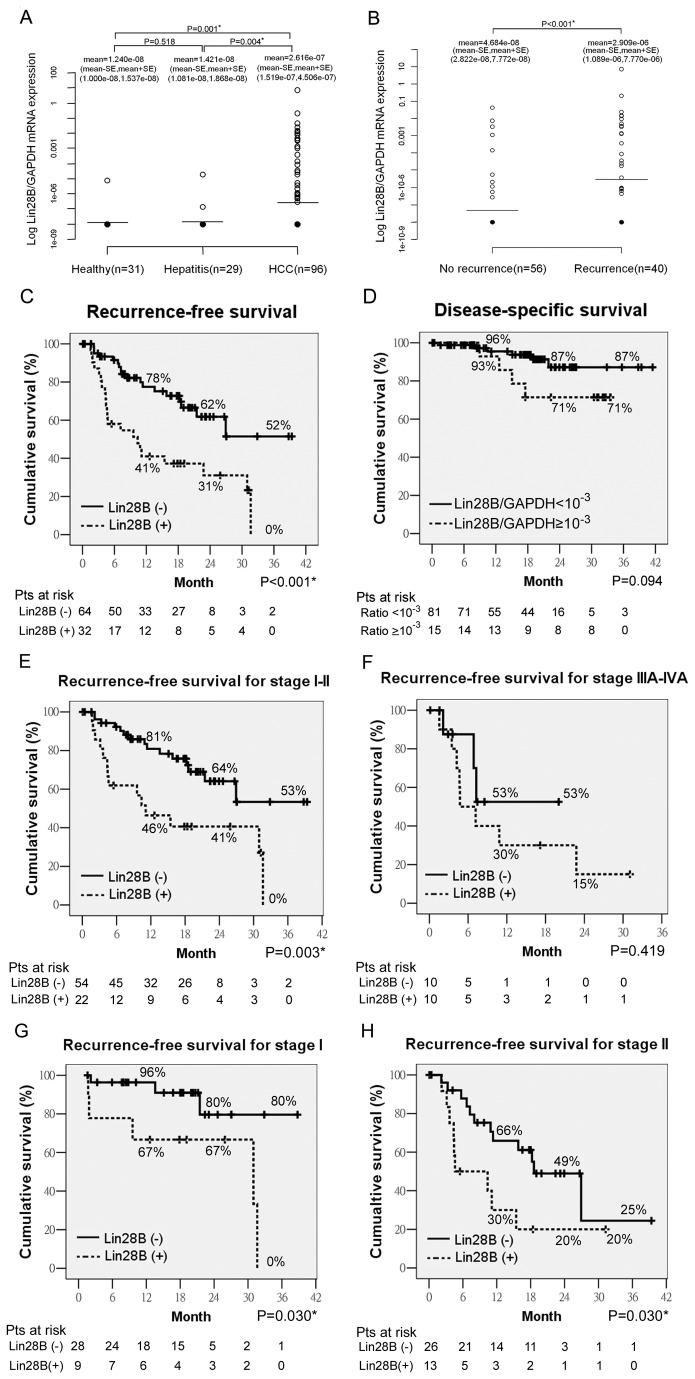
RT-qPCR quantification of the expression of *Lin28B* in peripheral blood monoculear cells. *Lin28B* was expressed in 3 cases (5%) in the non-HCC controls (1 in healthy group and 2 in hepatitis group) and in 32 cases (33.3%) in the HCC group. (Bar: mean; Black dot: undetectable) (**A**). Patients with recurrent HCC had significantly higher expression levels of *Lin28B* than patients without recurrent HCC. (P<0.001). (Bar: mean; Black dot: undetectable) (**B**). Kaplan-Meier analysis showed that *Lin28B* was significantly associated with decreased recurrence-free survival (P<0.001) (**C**). Ratio of *Lin28B*/*GAPDH* mRNA higher than 10^-3^ tended to associate with disease-specific survival (P=0.094) (**D**). *Lin28B* was significantly associated with decreased recurrence-free survival in AJCC stage I-II patients (P=0.003) (**E**) but not in AJCC stage IIIA-IVA patients (P=0.419) (**F**). *Lin28B* was significantly associated with decreased recurrence-free survival in AJCC stage I (P=0.030) (**G**) and stage II (P=0.030) patients (**H**).

 RT-qPCR was applied to examine the expression of *Lin28B* in the peripheral blood circulating cells. The reaction was linear from 10 to 10^6^ copies of purified plasmid templates (Figure S2). The expression of *Lin28B* could be detected when one HepG2 cell was pooled with 10^7^ leukocytes in about 3 ml of whole blood (Figure S5). On this base, *Lin28B* mRNA was detected in 3 cases (5%) of non-HCC controls (1 in healthy group and 2 in hepatitis group) and in 32 cases (33.3%) of HCC group (Figure 3A). The range of Ct was from 30.57 to 37.94 for the positive samples.

Patients with recurrent HCC had significantly higher expression levels of *Lin28B* than those without recurrence (P<0.001) (Figure 3B). The expression of *Lin28B* in circulating cells was significantly associated with non-cirrhotic liver (P=0.021), high tumor grade (P=0.046), large tumor size (P=0.005), high AJCC stage (P=0.044) and BCLC stage (P=0.017) (Table 1). The level of circulating Lin28B had a significant association with high BCLC stage (stage B to C diseases versus A1 to A4 diseases) (P=0.022) (Figure S6A) and had a borderline significance in associated with high AJCC stage (stage IIIC to IVA diseases versus I to IIIB diseases) (P=0.066) (Figure S6B). Kaplan-Meier analysis showed that circulating *Lin28B* was significantly associated with decreased RFS (P<0.001) (Figure 3C). The expression of *Lin28B* in circulating cells was not significantly associated with decreased DSS (P=0.140). By using a leave-one-out cross validation method, ratio of *Lin28B/GAPDH* mRNA higher than 10^-3^ (n=15) tended to be associated with decreased DSS (P=0.094) (Figure 3D). 

**Table 1:** Correlation of circulating *Lin28B* test results with clinicopathological indicators of hepatocellular carcinoma.

Factors	Group		*Lin28B* (−) (%)	*Lin28B* (+) (%)	*P*-value
Age	<60 years old		35 (70)	15 (30)	0.470
	≥60 years old		29 (63)	17 (37)	
Sex	Male		46 (67)	23 (33)	1.000
	Female		18 (67)	9 (33)	
Virus infection	None		10 (83)	2 (17)	0.551
	HBV		34 (61)	22 (39)	
	HCV		16 (67)	8 (33)	
	HBV+HCV		4 (100)	0 (0)	
Cirrhosis	Absent		28 (56)	22 (44)	0.021*
	Present		36 (78)	10 (22)	
Tumor grade	1-2		55 (71)	22 (29)	0.046*
	3		9 (47)	10 (53)	
Multifocal tumors	Absent		53 (65)	28 (35)	0.551
	Present		11 (73)	4 (27)	
Satellite nodule	Absent		51 (67)	25 (33)	0.859
	Present		13 (65)	7 (35)	
Tumor size	< 5 cm		45 (78)	13 (22)	0.005*
	≥ 5 cm		19 (50)	19 (50)	
Vascular invasion	Absent		35 (71)	14 (29)	0.312
	Present		29 (62)	18 (38)	
AJCC stage	I, II, IIIA, IIIB		62 (70)	27 (30)	0.044*
	IIIC, IVA		2 (29)	5 (71)	
BCLC stage	A1-A4		41 (77)	12 (23)	0.017*
	B-C		23 (53)	20 (47)	
Serum AFP levels	< 50 ng/ml		44 (73)	16 (27)	0.074
	≥ 50 ng/ml		20 (56)	16 (44)	

* P<0.05. Tumor grade by Edmondson and Steiner grading system. AJCC, American Joint Committee on Cancer 2010; BCLC, Barcelona-Clinic Liver Cancer; AFP, alpha-fetoprotein.

Univariate analysis revealed that tumor grade (P=0.047), vascular invasion (P=0.038), AJCC stage (P<0.001), BCLC stage (P=0.030) and circulating *Lin28B* (P=0.001) were significantly associated with decreased RFS (Table 2). In the multivariate model, cirrhosis (P=0.043), AJCC stage (P=0.001) and circulating *Lin28B* (P=0.047) were independent variables associated with decreased RFS (Table 2). Tumor grade (P=0.035), AJCC stage (P<0.001), serum AFP (P=0.014) and circulating *Lin28B* (P=0.001) were significantly associated with early recurrence less than one year in the univariate model (Table 3). AJCC stage (P=0.004) and circulating *Lin28B* (P=0.045) were significantly associated with early recurrence less than one year in the multivariate model (Table 3). As for DSS, satellite nodule (P=0.047) and AJCC stage (P=0.046) were independent variables in the multivariate analysis (Table S5). However, *Lin28B/GAPDH* mRNA higher than 10^-3^ was not an independent parameter in DSS. 

**Table 2:** Univariate and multivariate analyses of relation of circulating *Lin28B* and clinicopathological variables to recurrence-free survival in 96 patients with hepatocellular carcinoma.

	RFS univariate				RFS multivariate			
Factor	Group	HR	95% CI	P		HR	95% CI	P
Age	<60/≥60 years	0.647	(0.341-1.227)	0.182		0.470	(0.218-1.016)	0.055
Sex	Male/female	0.763	(0.371-1.569)	0.462		0.970	(0.439-2.140)	0.939
Viral infection				0.278				0.106
	None/B or C	2.840	(0.683-11.809)			6.186	(1.108-34.525)	
	None /Both	1.313	(0.118-14.566)			3.121	(0.201-48.354)	
Cirrhosis	-/+	0.717	(0.382-1.344)	0.299		0.419	(0.181-0.971)	0.043*
Tumor grade	1-2/3	2.081	(1.009-4.296)	0.047*		1.663	(0.631-4.379)	0.303
Multifocal tumor	-/+	1.644	(0.722-3.745)	0.236		3.038	(0.991-9.317)	0.052
Satellite nodule	-/+	1.803	(0.889-3.654)	0.102		1.965	(0.765-5.047)	0.161
Tumor size	<5/≥5 cm	1.556	(0.828-2.925)	0.170		0.503	(0.117-2.169)	0.357
Vascular invasion	-/+	1.961	(1.039-3.703)	0.038*		1.182	(0.433-3.230)	0.744
AJCC stage				<0.001*				0.001*
	I/II~IIIB	3.421	(1.396-8.385)			4.929	(1.293-18.785)	
	I/IIIC~IVA	36.35	(3.731-353.236)			50.281	(4.202-601.720)	
BCLC stage				0.030*				0.085
	A1/A2-A4	2.986	(1.209-7.374)			3.632	(1.073-12.288)	
	A1/B-C	2.361	(1.124-4.961)			3.320	(0.730-15.110)	
Serum AFP	<50/≥50 ng/ml	1.642	(0.876-3.076)	0.122		0.778	(0.323-1.873)	0.575
Lin28B	-/+	2.918	(1.559-5.463)	0.001*		2.248	(1.012-4.995)	0.047*

* P < 0.05. Tumor grade by Edmondson and Steiner grading system. AJCC, American Joint Committee on Cancer 2010; BCLC, Barcelona-Clinic Liver Cancer; AFP, alpha-fetoprotein. RFS, Recurrence-free survival.

**Table 3:** Univariate and multivariate analyses of relation of circulating *Lin28B* and clinicopathological variables to recurrence-free survival less than one year in 96 patients with hepatocellular carcinoma.

	RFS<1year univariate				RFS<1 year multivariate			
Factor	Group	HR	95% CI	P		HR	95% CI	P
Age	<60/≥60 years	0.616	(0.293-1.295)	0.201		0.479	(0.189-1.215)	0.121
Sex	Male/female	0.570	(0.233-1.396)	0.219		0.742	(0.276-2.000)	0.556
Viral infection				0.528				0.315
	None/B or C	2.080	(0.494-8.760)			4.314	(0.653-28.510)	
	None /Both	1.152	(0.104-12.724)			2.777	(0.156-49.508)	
Cirrhosis	-/+	0.576	(0.104-12.724)	0.147		0.437	(0.153-1.250)	0.123
Tumor grade	1-2/3	2.320	(1.061-5.074)	0.035*		1.751	(0.571-5.362)	0.327
Multifocal tumor	-/+	1.755	(0.714-4.312)	0.220		3.071	(0.882-10.689)	0.078
Satellite nodule	-/+	1.454	(0.647-3.267)	0.365		1.759	(0.590-5.245)	0.311
Tumor size	<5/≥5 cm	1.996	(0.973-4.095)	0.059		0.632	(0.131-3.055)	0.568
Vascular invasion	-/+	1.847	(0.889-3.838)	0.100		0.871	(0.281-2.704)	0.811
AJCC stage				<0.001*				0.004*
	I/II~IIIB	3.782	(1.521-9.405)			4.040	(0.948-17.221)	
	I/IIIC~IVA	37.631	(3.860-366.894)			47.592	(3.692-613.500)	
BCLC stage				0.063				0.304
	A1/A2-A4	2.015	(0.639-6.351)			2.532	(0.606-10.582)	
	A1/B-C	2.856	(1.190-6.850)			2.787	(0.536-14.502)	
Serum AFP	<50/≥50 ng/ml	2.457	(1.198-5.038)	0.014*		0.964	(0.344-2.698)	0.944
Lin28B	-/+	3.637	(1.749-7.563)	0.001*		2.649	(1.022-6.862)	0.045*

* P < 0.05. Tumor grade by Edmondson and Steiner grading system. AJCC, American Joint Committee on Cancer 2010; BCLC, Barcelona-Clinic Liver Cancer; AFP, alpha-fetoprotein. RFS, Recurrence-free survival.

If further stratified by AJCC stage, circulating *Lin28B* significantly correlated with RFS in earlier stages (stage I and stage II) (P=0.003) (Figure 3E) although it did not reach statistical significance in patients with more advanced stages (P=0.419) (Figure 3F). Circulating *Lin28B* still significantly correlated with RFS in stage I and stage II patients, separately (P=0.030 and P=0.030, respectively) (Figure 3G and 3H). In multivariate analysis, circulating *Lin28B* (P=0.006) and AJCC stage (P=0.023) were independent variables associated with decreased RFS in earlier stages (Table S6). 

## Discussion

In this study we found that the oncofetal cancer-stem-cell-like marker, *Lin28B* in peripheral blood mononuclear cells is associated with early recurrence of HCC. Moreover, our result showed that circulating *Lin28B* can separate early stage HCC into 2 RFS curves. Such a phenomenon was not reported in previous studies. 

Several recent studies reported that circulating cancer stem cells in general correlate well with tumor recurrence and poor prognosis [21,22,31]. Theoretically, CTCs may comprise differentiated tumor cells and/or cancer stem cells. Differentiated CTCs might have limited or no proliferation capabilities, frequently exhibit apoptosis and are not likely to establish a metastatic lesion at distant sites [32]. On the contrary, circulating cancer stem cells could regenerate the entire population of tumor cells at metastatic sites [33,34]. In HCCs, circulating cancer stem cells may also homing back to liver and thus contribute to early tumor recurrence. More importantly, the prediction power for recurrence observed in this study is independent of the TNM stages. We could refine stage I and stage II patients into different recurrence curves by the status of circulating *Lin28B*. It has been proposed to use CTC in the modification of the staging system in breast cancer [35]. Our finding supports the possible use of a TNMC (C for CTC) staging system in HCC. The predictive value in advanced HCC needs to be further evaluated due to small case number (n=20) in this subgroup.

In this study, we demonstrated the expression of *Lin28B* in a stem cell-like subpopulation in HCC cell lines and the potential of *Lin28B* in inducing the stem-like characteristics of HCC. The results concur with recent studies showing that enforced *Lin28B* expression induces the expression of stem cell-related genes in multiple cell types [5,14,15]. Thus, *Lin28B* may play a certain role in inducing or maintaining cancer stem cell phenotypes. In addition, *Lin28B* could up-regulate the let-*7* target genes, including *Myc, HMGA2*, and *IGF1R* [5,6,11]. The findings may explain the effect of *Lin28B* on cell proliferation, epithelial to mesenchymal transition, invasion and tumorigenesis *in vitro*, as well as the high tumor grading and early tumor recurrence [9-11]. We found that the detection of circulating *Lin28B* is also associated with high tumor grade, large tumor size and high AJCC stage and BCLC stage. The level of circulating *Lin28B* is significantly higher in high BCLC stages. Interestingly, circulating *Lin28B* was also associated with non-cirrhotic status of HCC patients. Since there was low level of *Lin28B* expression in only two out of 15 (13%) non-tumor liver tissue samples and one of these two samples showed cirrhosis, this association may not be due to non-cirrhotic liver status itself. It is most likely due to significantly larger tumor size (P=0.001) and borderlinely higher BCLC stage (P=0.067) in non-cirrhotic HCC patients compared with cirrhotic HCC patients (data not shown). Taken together, circulating *Lin28B* could serve as a prognostic marker. Future study is needed to evaluate the potential of circulating *Lin28B* in screening or early diagnosis of HCC.

The US Food and Drug Administration–approved CellSearch system employs the anti-EpCAM and anti-cytokeratin antibodies to select the CTCs and anti-CD45 antibody to exclude the possibility of lymphocytes. The CellSearch system has been demonstrated to be a predictive marker for survival in breast cancer [36]. EpCAM was also reported as a surface marker for stem/progenitor cells of liver and is associated with a more aggressive behavior in HCC patients [28,37]. Therefore in theory, EpCAM could also be a good surface marker for circulating HCC stem cells. However, we failed to substantiate the stemness nature of EpCAM in HepG2 cell line. This observation seems reasonable considering the fact that cancer stem cells may associate with epithelial-mesenchymal transition with down-regulation of epithelial markers, such as EpCAM and cytokeratin [34,38]. We propose that EpCAM alone may not be sufficient in the identification of HCC stem cells and that *Lin28B* could be a novel HCC stem cell marker, in addition to CD133, CD90, CD44, OVA6, ALDH, and CD13 [39]. Given that circulating CD45^-^CD90^+^CD44^+^ cells detected by flow cytometry were associated with early HCC recurrence, circulating cancer stem cell markers may have significant prognostic values in HCC [21]. However, RT-qPCR measurement of *Lin28B* may have the advantages of simplicity and higher sensitivity than the cell based analyses. More importantly, the oncofetal character of *Lin28B* may be advantageous over the other markers. For example, CD133 is expressed in the endothelial cells and thus is unreliable as a marker for circulating cancer stem cells [40]. Further survey will be needed to determine if adding *Lin28B* in a panel of cancer stem cell markers will increase the prognostic significance.

There are limitations to this study. First, although *Lin28B* was more often detected in the circulating cells of HCC patients (33.3%), it was detected in those of non-HCC controls (5%). This result is in line with *AFP*, the prototype of oncofetal genes/proteins. *AFP* mRNA in circulating cells had been detected in 8-72.7% of patients with HCC[41-45] and in 3.3% to 17% of controls without HCC[45,46]. Plausible explanation for the detection in controls includes the exfoliation of non-malignant regenerate hepatocytes into circulation due to liver inflammation, illegitimate transcription within leukocytes, or actual presence of minimal malignant cells [46]. The first possibility is reasonable since two of 13 non-tumor liver tissue samples (13%) showed low level of *Lin28B* expression. Further prospective study is needed to clarify the prognostic value of measuring circulating *Lin28B* in patients with chronic hepatitis. Second, although we found that circulating *Lin28B* had a trend correlating with disease-specific survival, the statistical result was not significant. This may be due to relative early stage (79.2% of cases were in AJCC stage I or II) and short follow-up of the patients. A long-standing follow-up study on larger cohort with more advanced stage and different treatment modalities is required to confirm the overall prognostic significance of *Lin28B* RT-qPCR test.

In conclusion, we identified *Lin28B* as a potential circulating oncofetal cancer-stem-cell-like marker in predicting the recurrence of HCC. *Lin28B* RT-qPCR test of peripheral blood samples may refine the current staging system and be used to select a subset of HCC patients for more intensive post-operative follow-up and chemoprevention protocol. Furthermore, our finding may also lead to the recognition of other oncofetal cancer-stem-cell-like markers for the circulating cells with clinical significance.

## Supporting Information

Figure S1 **The participant flow diagram.**
(TIF)

Figure S2 **The performance of the *Lin28B* RT-qPCR.** The reaction was linear from 10 to 10^6^ copies of purified plasmid templates. The standard curve was calculated using LightCycler 4.05 software. The reaction efficiency was between 1.8 and 2.0.
(TIF)

Figure S3 **RT-PCR analysis of *Lin28B* expression in various tumor cell lines.** *Lin28B* was expressed in ovarian (PA-1, BG1, OVCAR-3, and ES-2), hepatic (Hu-h7, HepG2), and colon (SW480) tumor cell lines. β-actin was the internal control.
(TIF)

Figure S4 **RT-PCR analysis of *Lin28B* expression in tumor/non-tumor tissue pairs.** *Lin28B* was expressed in breast (1/5), uterine (1/5), pulmonary (4/5), hepatic (1/5), and ovarian (1/5) tumor tissue, but not in the non-tumor tissue. β-actin was the internal control. (N = non-tumor, T = tumor).
(TIF)

Figure S5 **The detection limit of *Lin28B* RT-qPCR for the spiked HCC cancer cells.** HepG2 cells (1-10^5^) were pooled into 10^7^ peripheral blood leukocytes (PBLs, about 3 ml of whole blood) and analyzed by RT-qPCR. The detection limit was one HepG2 cell in 10^7^ PBLs.
(TIF)

Figure S6 **A**. Patients with higher BCLC stage had significantly higher expression levels of *Lin28B* (P=0.022). **B**. Patients with higher AJCC stage had a borderline higher expression levels of *Lin28B* (P=0.066). (Bar: mean; Black dot: undetectable).
(TIF)

Table S1 **Frequencies of *Lin28B* gene in immature, mature and tumor groups of the expressed sequence tag (EST) libraries compared with *AFP* and *LRRC16B*.**
(DOCX)

Table S2 **Patient profiles.**
(DOCX)

Table S3 **Primer sequences for reverse-transcription polymerase chain reactions.**
(DOCX)

Table S4 **Primer and probe sequences for real-time quantitative polymerase chain reactions.**
(DOCX)

Table S5 **Univariate and multivariate analyses of relation of circulating *Lin28B* and clinicopathological variables to disease-specific survival in 96 patients with hepatocellular carcinoma.**
(DOCX)

Table S6 **Univariate and multivariate analyses of relation of circulating *Lin28B* and clinicopathological variables to recurrence-free survival in 76 patients with AJCC stage I and II hepatocellular carcinoma.**
(DOCX)
