# Supplementary figures and images for: Lin28B Is an Oncofetal Circulating Cancer Stem Cell-Like Marker Associated with Recurrence of Hepatocellular Carcinoma
Source: PLoS One. 2013 Nov 14;8(11):e80053. doi: 10.1371/journal.pone.0080053 (PMC3828221; doi:10.1371/journal.pone.0080053)

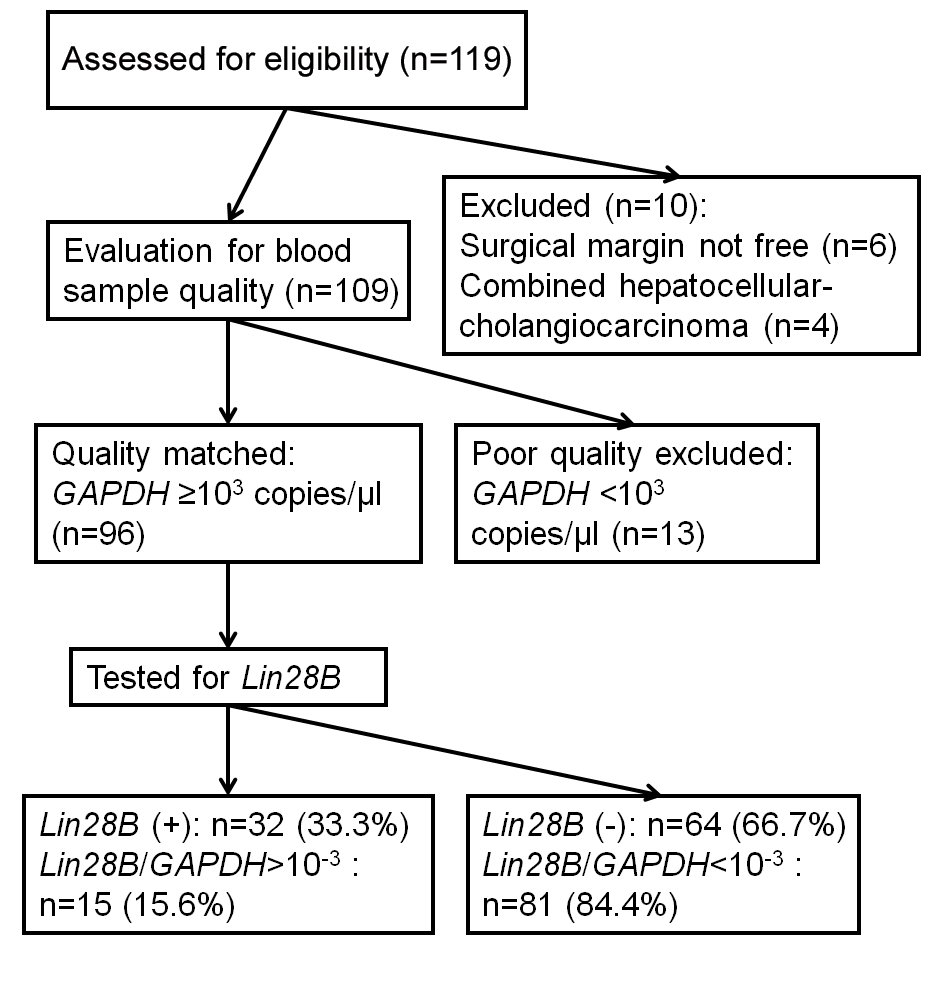

Supplement: Figure S1 — The participant flow diagram. (TIF) [file pone.0080053.s001.tif]

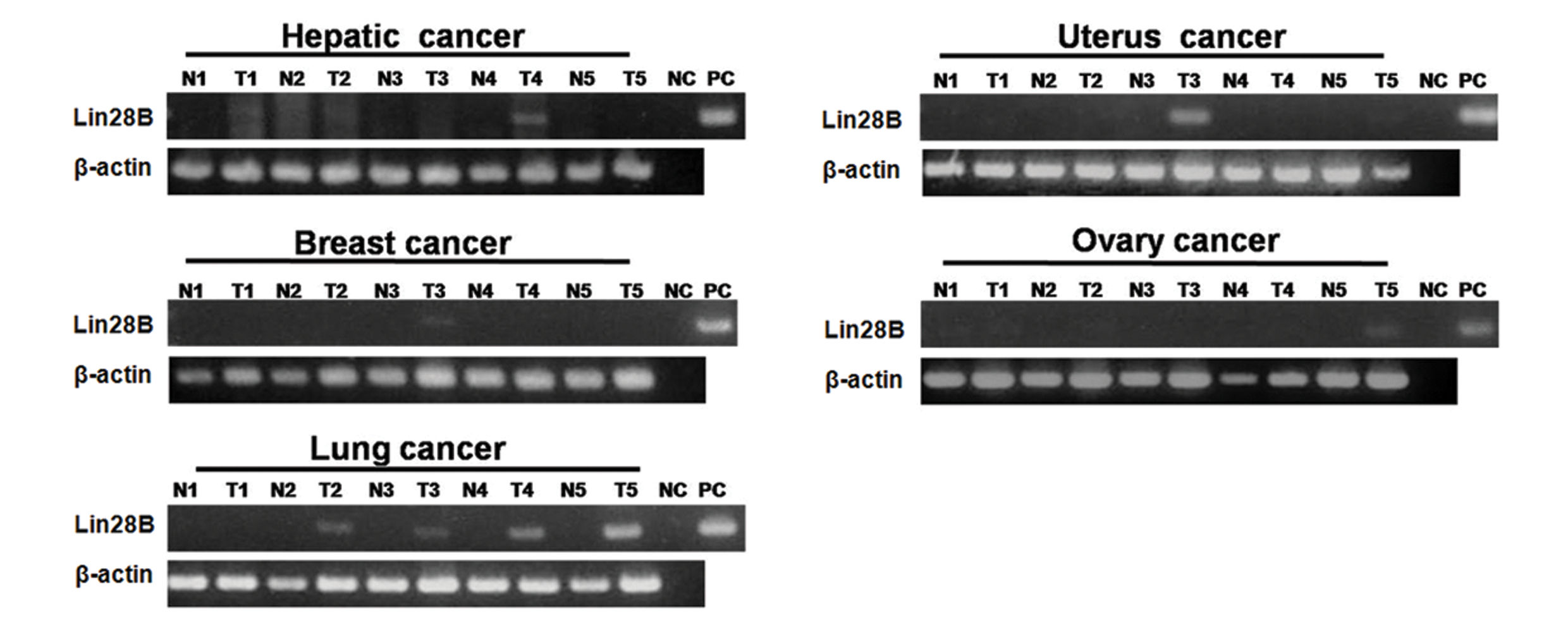

Supplement: Figure S4 — RT-PCR analysis of Lin28B expression in tumor/non-tumor tissue pairs. Lin28B was expressed in breast (1/5), uterine (1/5), pulmonary (4/5), hepatic (1/5), and ovarian (1/5) tumor tissue, but not in the non-tumor tissue. β-actin was the internal control. (N = non-tumor, T = tumor). (TIF) [file pone.0080053.s004.tif]

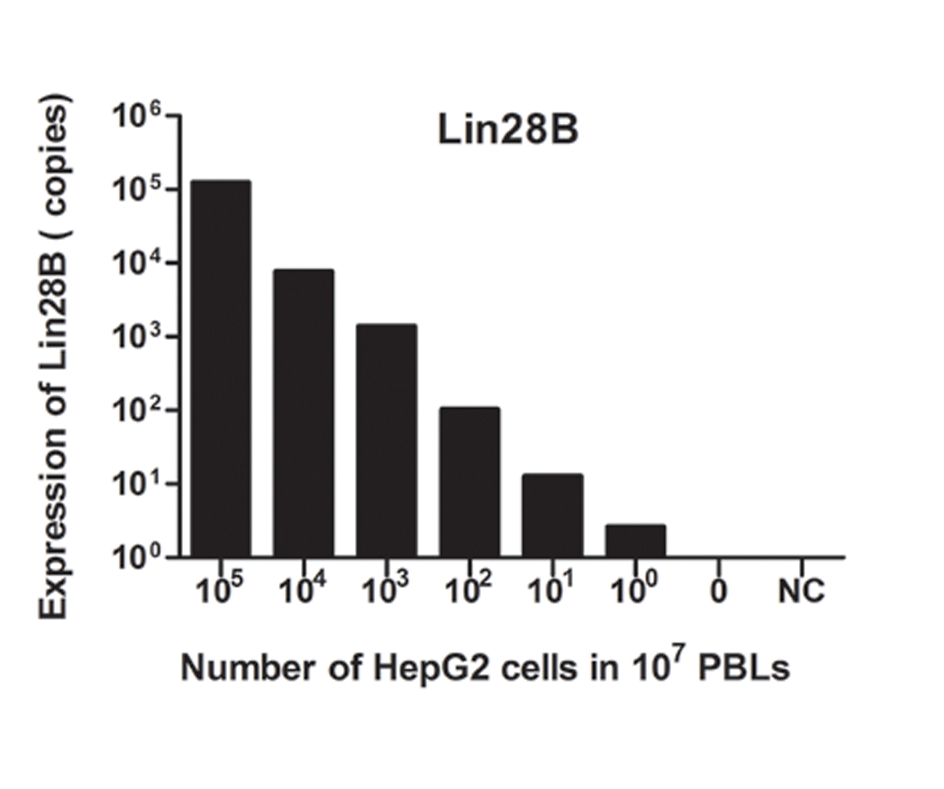

Supplement: Figure S5 — The detection limit of Lin28B RT-qPCR for the spiked HCC cancer cells. HepG2 cells (1-105) were pooled into 107 peripheral blood leukocytes (PBLs, about 3 ml of whole blood) and analyzed by RT-qPCR. The detection limit was one HepG2 cell in 107 PBLs. (TIF) [file pone.0080053.s005.tif]

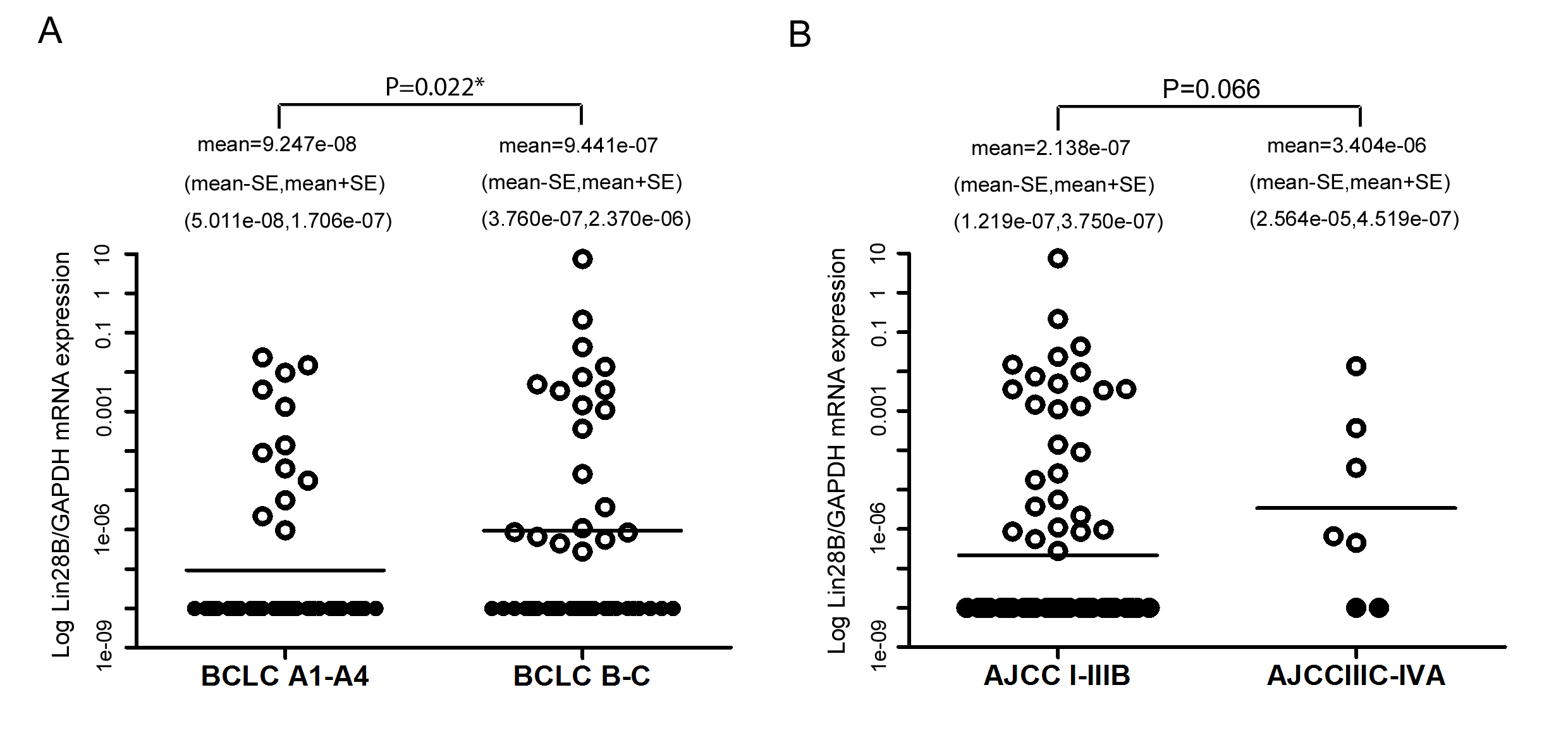

Supplement: Figure S6 — A. Patients with higher BCLC stage had significantly higher expression levels of Lin28B (P=0.022). B. Patients with higher AJCC stage had a borderline higher expression levels of Lin28B (P=0.066). (Bar: mean; Black dot: undetectable). (TIF) [file pone.0080053.s006.tif]
