# Supplementary material for: Lin28B Is an Oncofetal Circulating Cancer Stem Cell-Like Marker Associated with Recurrence of Hepatocellular Carcinoma
Source: PLoS One. 2013 Nov 14;8(11):e80053. doi: 10.1371/journal.pone.0080053 (PMC3828221; doi:10.1371/journal.pone.0080053)
Supplement: Table S1 — Frequencies of Lin28B gene in immature, mature and tumor groups of the expressed sequence tag (EST) libraries compared with AFP and LRRC16B. (DOCX) [file pone.0080053.s007.docx]

Table S1. Frequencies of *Lin28B* gene in immature, mature and tumor groups of the expressed sequence tag (EST) libraries compared with *AFP* and *LRRC16B.*

| Gene | Immature | Mature | Tumor |
| --- | --- | --- | --- |
| *AFP* | 175 | 10 | 108 |
| *LRRC16B* | 9 | 0 | 15 |
| *Lin28B* | 22 | 5 | 28 |
