# Supplementary material for: Lin28B Is an Oncofetal Circulating Cancer Stem Cell-Like Marker Associated with Recurrence of Hepatocellular Carcinoma
Source: PLoS One. 2013 Nov 14;8(11):e80053. doi: 10.1371/journal.pone.0080053 (PMC3828221; doi:10.1371/journal.pone.0080053)
Supplement: Table S2 — Patient profiles. (DOCX) [file pone.0080053.s008.docx]

Table S2. Patient profiles.

| Variables |  |
| --- | --- |
| Age (years): mean, range | 59.13, 27~86 |
| Sex: male / female (cases) | 69 / 27 |
| Hepatitis virus (cases): B / C / B+C/ Non-B Non-C | 56 / 24 / 4 / 12 |
| Alpha-fetoprotein (ng/ml): median, range | 14.96, 0.88~68850 |
| Cirrhosis (cases): no / yes | 50 / 46 |
| Tumor size (cm): Mean, range | 5.35, 1~17 |
| Tumor grade (cases): 1 / 2 / 3 | 14 / 63 / 19 |
| Satellite nodule (cases): no / yes | 76 / 20 |
| Multifocal tumor (cases): no / yes | 81 / 15 |
| Vascular invasion (cases): no / microscopic / major branches | 49 / 43 / 4 |
| AJCC stage (cases): I / II / IIIA / IIIB / IIIC / IVA | 37 / 39 / 10 / 3 / 6 / 1 |
| BCLC stage (cases): A1/A2/A3/A4/B/C | 38 / 5 / 1 / 9 / 36 / 7 |

Tumor grade by Edmondson and Steiner grading system. AJCC, American Joint Committee on Cancer 2010; BCLC, Barcelona-Clinic Liver Cancer.
