# Supplementary material for: Lin28B Is an Oncofetal Circulating Cancer Stem Cell-Like Marker Associated with Recurrence of Hepatocellular Carcinoma
Source: PLoS One. 2013 Nov 14;8(11):e80053. doi: 10.1371/journal.pone.0080053 (PMC3828221; doi:10.1371/journal.pone.0080053)
Supplement: Table S3 — Primer sequences for reverse-transcription polymerase chain reactions. (DOCX) [file pone.0080053.s009.docx]

Table S3. Primer sequences for reverse-transcription polymerase chain reactions.

| Gene | Primer Sequence |
| --- | --- |
| *Lin28B* (forward) | 5’-CCTTGAGTCAATACGGGT-3’ |
| *Lin28B* (reverse) | 3’-GCTCTGACAGTAATGGCA-5’ |
| *β-actin* (forward) | 5’-CATGTACGTTGCTATCCAGGC-3’ |
| *β-actin* (reverse) | 3’-CTCCTTAATGTCACGCACGAT-5’ |
