# Supplementary material for: Lin28B Is an Oncofetal Circulating Cancer Stem Cell-Like Marker Associated with Recurrence of Hepatocellular Carcinoma
Source: PLoS One. 2013 Nov 14;8(11):e80053. doi: 10.1371/journal.pone.0080053 (PMC3828221; doi:10.1371/journal.pone.0080053)
Supplement: Table S4 — Primer and probe sequences for real-time quantitative polymerase chain reactions. (DOCX) [file pone.0080053.s010.docx]

Table S4. Primer and probe sequences for real-time quantitative polymerase chain reactions.

| Gene* | Primer Sequence |
| --- | --- |
| *Lin28B* (Probe) | 5’-FAM-CATGATGATCAAGGCCACCACAGT-BHQ-3’ |
| *Lin28B* (Forward primer) | 5’-ACCCAAAGGGAAGACACTACAG-3’ |
| *Lin28B* (Reverse primer) | 3’-TTTGGCTGAGGAGGTAGACTAC-5’ |
| *PLA* (Probe) | 5’-FAM-CATCACCAGCGGCAACCTCA-BHQ-3’ |
| *PLA* (Forward primer) | 5’-ACAAGCAGAGAGCAAAGTCTTC-3’ |
| *PLA* (Reverse primer) | 3’-GTTGTGACTGATCGACAATCCC-5’ |

*The probes and probe sequences of *GAPDH* is not shown.
