# Supplementary material for: Lin28B Is an Oncofetal Circulating Cancer Stem Cell-Like Marker Associated with Recurrence of Hepatocellular Carcinoma
Source: PLoS One. 2013 Nov 14;8(11):e80053. doi: 10.1371/journal.pone.0080053 (PMC3828221; doi:10.1371/journal.pone.0080053)
Supplement: Table S5 — Univariate and multivariate analyses of relation of circulating Lin28B and clinicopathological variables to disease-specific survival in 96 patients with hepatocellular carcinoma. (DOCX) [file pone.0080053.s011.docx]

Table S5. Univariate and multivariate analyses of relation of circulating *Lin28B* and clinicopathological variables to disease-specific survival in 96 patients with hepatocellular carcinoma.

|  | DSS univariate | | | | DSS multivariate | | | |
| --- | --- | --- | --- | --- | --- | --- | --- | --- |
| Factor | Group | HR | 95% CI | P |  | HR | 95% CI | P |
| Age | <60/≥60 years | 0.806 | (0.227-2.860) | 0.739 |  | 0.581 | (0.053-6.389) | 0.657 |
| Sex | Male/female | 0.982 | (0.254-3.803) | 0.979 |  | 8.607 | (0.677-109.351) | 0.097 |
| Viral infection |  |  |  | 0.318 |  |  |  | 0.385 |
|  | None/B or C | 0.327 | (0.067-1.585) |  |  | 5.558 | (0.070-439.561) |  |
|  | None /Both | 0.790 | (0.071-8.774) |  |  | 46.526 | (0.164-13208.512) |  |
| Cirrhosis | -/+ | 0.418 | (0.108-1.617) | 0.206 |  | 0.142 | (0.017-1.208) | 0.074 |
| Tumor grade | 1-2/3 | 4.860 | (1.406-16.804) | 0.012* |  | 0.176 | (0.013-2.306) | 0.186 |
| Multifocal tumor | -/+ | 2.909 | (0.751-11.273) | 0.122 |  | 13.823 | (0.932-205.061) | 0.056 |
| Satellite nodule | -/+ | 4.010 | (1.160-13.869) | 0.028* |  | 13.422 | (1.040-173.155) | 0.047* |
| Tumor size | <5/≥5 cm | 8.229 | (1.743-38.840) | 0.008* |  | 74.456 | (0.674-8221.940 | 0.073 |
| Vascular invasion | -/+ | 4.394 | (0.933-20.699) | 0.061 |  | 0.657 | (0.032-13.399) | 0.784 |
| AJCC stage | I-IIIA/IIIB-IVA | 14.500 | (4.115-51.089) | ＜0.001* |  | 41.430 | (1.061-1618.362 | 0.046* |
| BCLC stage | A1-A4/B-C | 13.193 | (1.669-104.303) | 0.014* |  | 0.217 | (0.002-28.791) | 0.540 |
| Serum AFP | <50/≥50 ng/ml | 18.344 | (2.321-144.969) | 0.006* |  | 6.999 | (0.320-153.056) | 0.216 |
| Lin28B ratio>10^-3^ | -/+ | 2.832 | (0.794-10.103) | 0.109 |  | 5.995 | (0.515-69.721) | 0.153 |

^*^P < 0.05. Tumor grade by Edmondson and Steiner grading system. AJCC, American Joint Committee on Cancer 2010; BCLC, Barcelona-Clinic Liver Cancer; AFP, alpha-fetoprotein.
