# Supplementary material for: Lin28B Is an Oncofetal Circulating Cancer Stem Cell-Like Marker Associated with Recurrence of Hepatocellular Carcinoma
Source: PLoS One. 2013 Nov 14;8(11):e80053. doi: 10.1371/journal.pone.0080053 (PMC3828221; doi:10.1371/journal.pone.0080053)
Supplement: Table S6 — Univariate and multivariate analyses of relation of circulating Lin28B and clinicopathological variables to recurrence-free survival in 76 patients with AJCC stage I and II hepatocellular carcinoma. (DOCX) [file pone.0080053.s012.docx]

Table S6. Univariate and multivariate analyses of relation of circulating *Lin28B* and clinicopathological variables to recurrence-free survival in 76 patients with AJCC stage I and II hepatocellular carcinoma.

|  | RFS univariate | | | | RFS multivariate | | | |
| --- | --- | --- | --- | --- | --- | --- | --- | --- |
| Factor | Group | HR | 95% CI | P |  | HR | 95% CI | P |
| Age | <60/≥60 years | 0.627 | (0.317-1.426) | 0.300 |  | 0.496 | (0.194-1.264) | 0.142 |
| Sex | Male/female | 0.680 | (0.288-1.603) | 0.378 |  | 0.848 | (0.328-2.191) | 0.733 |
| Viral infection | None, B or C/Both | 0.614 | (0.083-4.545) | 0.633 |  | 0.895 | (0.079-10.080) | 0.928 |
| Cirrhosis | -/+ | 0.842 | (0.404-1.753) | 0.645 |  | 0.661 | (0.261-1.678) | 0.384 |
| Tumor grade | 1-2/3 | 1.396 | (0.482-4.043) | 0.538 |  | 2.514 | (0.630-10.040) | 0.192 |
| Multifocal tumor | -/+ | 2.077 | (0.711-6.063) | 0.181 |  | 1.265 | (0.176-9.079) | 0.815 |
| Satellite nodule | -/+ | 2.102 | (0.882-5.006) | 0.093 |  | 1.233 | (0.307-4.954) | 0.768 |
| Tumor size | <5/≥5 cm | 1.289 | (0.568-2.926) | 0.544 |  | 0.541 | (0.073-4.032) | 0.549 |
| Vascular invasion | -/+ | 1.478 | (0.712-3.066) | 0.295 |  | 0.514 | (0.126-2.094) | 0.353 |
| AJCC stage | I/II | 3.275 | (1.441-7.444) | 0.005* |  | 7.293 | (1.316-40.428) | 0.023* |
| BCLC stage | A1-A4/B-C | 1.464 | (0.688-3.116) | 0.322 |  | 1.182 | (0.224-6.244) | 0.844 |
| Serum AFP | <50/≥50 ng/ml | 0.921 | (0.408-2.082) | 0.844 |  | 0.538 | (0.207-1.398) | 0.204 |
| Lin28B positive | -/+ | 2.873 | (1.378-5.988) | 0.005* |  | 3.308 | (1.417-7.722) | 0.006* |

^*^P < 0.05. Tumor grade by Edmondson and Steiner grading system. AJCC, American Joint Committee on Cancer 2010; BCLC, Barcelona-Clinic Liver Cancer; AFP, alpha-fetoprotein.
